# Decreased Cardiac NOX4 and SIRT-1 Protein Levels Contribute to Decreased Angiogenesis in the Heart of Diabetic Rats: Rescue Effects of IGF-1 and Exercise

**DOI:** 10.34172/apb.2023.039

**Published:** 2022-01-03

**Authors:** Shiva Roshan Milani, Bagher Pourheydar, Saman Daneshfar, Leila Chodari

**Affiliations:** ^1^Department of Physiology, Faculty of Medicine, Urmia University of Medical Sciences, Urmia, Iran.; ^2^Neurophysiology Research Center, Cellular and Molecular Medicine Institute, Urmia University of Medical Sciences, Urmia, Iran.; ^3^Department of Anatomical Sciences, School of Medicine, Urmia University of Medical Sciences, Urmia, Iran.; ^4^Faculty of Medicine, Urmia University of Medical Sciences, Urmia, Iran.

**Keywords:** Diabetes, Heart, Angiogenesis, Exercise, IGF-I, NOX4, SIRT1

## Abstract

***Purpose:*** Reduced angiogenesis in the heart tissue is a primary risk factor for heart disease in the diabetes condition. This study was aimed to evaluate the changes of two main angiogenesis mediators, NADPH oxidase 4 (NOX4) and sirtuin 1 (SIRT-1) protein levels in the heart of diabetic rats and the impact of Insulin-like growth factor 1 (IGF-1) and exercise on these proteins.

***Methods:*** Injection of 60 mg/kg of streptozotocin in 40 male Wistar rats led to the induction of type 1 diabetes. Angiogenesis was detected in the hearts by immunostaining for PECAM-1/ CD31 after 30 days of treatment with IGF-1 (2 mg/kg/day) and exercise. ELISA technique was utilized to establish the expression levels of NOX4 and SIRT-1 within the heart.

***Results:*** The results revealed a significant increase in HbA1c and a significant decrease in SIRT1, NOX4 levels and angiogenesis grade in the heart of diabetes group compared to control group. Meanwhile, IGF-1 and exercise alone or in combination completely masked these effects. Additionally, synergistic effect on SIRT-1, HbA1c levels and angiogenesis grade is evident when IGF-1 and exercise are applied simultaneously.

***Conclusion:*** Our findings suggest that reduction in angiogenesis in the heart of diabetic rats may be mediated by down expression of NOX4 and SIRT-1 protein levels. It was also displayed that IGF-1 and exercise as novel therapies increase NOX4 and SIRT-1 protein levels within the hearts of diabetic rats.

## Introduction

 Diabetes mellitus is a considerable public health problem and has a global impact on human health and economics.^1^ High blood glucose condition in the diabetes induces macro and microvascular malfunction and lowers angiogenesis in the cardiac tissue that leads to ischemic condition.^2^ Today, it is well known that decreased angiogenesis is a main reason for cardiac failure in diabetic patient.^2^ It has been demonstrated that diabetes impairs collateral vessel development in animal models of ischemia and in ischemic human hearts.^3^

 As a result, evaluating the molecular intermediators involved in the angiogenesis signaling pathways in diabetic rats’ heart tissue are critical. Several lines of evidence demonstrated that NOX4 (NADPH oxidase 4) and SIRT-1 (sirtuin 1) have significant impact on angiogenesis signaling pathway.^4-6^

 reactive oxygen species (ROS) scavenging enzymes and ROS producing oxidases cautiously co-regulate angiogenesis. The NOX family, by transferring electrons from NADPH to molecular oxygen produce ROS.^7^

 NOX4 is the key isoform of NADPH oxidases produced in endothelium and largely yields ROS and H2O2 that play crucial role in vascularization.^8^ It is shown that vascularization in cultured NOX4 ^-/-^ endothelial cells was reduced whereas treatment with H2O2 improve formation of new vessels.^9^

 Vasoprotective feature of NOX4 during ischemic also reported, furthermore molecular studies indicate that NOX4 stimulates angiogenesis signaling pathway through production of ROS.^10^ Although NOXs have been suggested as a therapeutic pathway for a variety of diabetic issues, their role in diabetic cardiomyopathy is still vague.^11^

 Sirtuins (Sirt1–Sirt7) include a family of nicotinamide adenine dinucleotide (NAD + )-dependent deacetylating enzymes.^12^ Between sirtuins, SIRT1 and SIRT6 are the best considered for their defensive role in opposition to inflammation, vascular and cardiac disease.^13^ SIRT1 is extremely produced in the vascular bed, where it controls endothelial angiogenic functions during vascular growth.^14^ In the absence of SIRT1 activity, the sprouting and branching blood vessels reduced.^15^ It is demonstrated that lowered expression of SIRT1 within the heart of diabetic subjects is enough to cause phenotypes like Diabetic cardiomyopathy.^16^

 Insulin-like growth factor 1 (IGF-1) as an inducer of angiogenesis factor is a peptide hormone that increases cell responsiveness to insulin.^17^ Numerous literatures have confirmed that in type 1 diabetes, IGF-1 insufficiency is prevalent and therefore sensitivity of cells to insulin decline in the diabetes condition.^18^ Furthermore, it is shown that IGF-1 therapy refines insulin sensitivity in adults with type 1 diabetes.^19^ Therefore, IGF-1 can be a useful healing goal in the treatment of heart failure in diabetes condition.

 In patients with heart disease, physical exercise is agreed to be one of the mending factor since it decrease myocardial failure, and cardiac risk factors, and enhances cardiac function.^20^ Regular exercise is known to decrease cardiovascular problems in both type 1 and 2 diabetes.^21^ Huge studies showed that exercise alleviates diabetes-induced cardiomyopathy by increasing angiogenesis.^22^

 Thus, this research aimed to study the effect of diabetes on cardiac NOX4 and SRIT-1 protein levels as antigenic factors. It also sought to examine the possible protective effects of treatments, alone or together, on angiogenesis in the heart in the diabetes condition and its corresponding molecular mediators (NOX4 and SIRT-1)

## Materials and Methods

###  Animals and study design

 Forty male Wistar rats weighing 250 ± 10 purchased from the Urmia University of Medical Science that were casually separated into 5 groups (n = 8). Animals were housed in special cages on a 12L:12D cycle at 24°C room temperature, proportionate moisture of 50% and fed ad libitum on commercial laboratory food pellets. The groupings are as follows: Control (Cont), Diabetes (Dia), Diabetes + Exercise (Dia + Exe), Diabetes + IGF-1 (Dia + IGF-1), Diabetes Exercise + IGF-1 (Dia + Exe + IGF-1) groups. IGF-1 (Sigma Aldrich, USA) was injected daily with 2 mg/kg dose subcutaneously for four weeks.^23^ It should be noted that rats that didn’t performed exercise were taken to the exercise room and placed on treadmill. Also, rats without IGF-1 intervention received normal saline daily.

###  Induction of type 1 diabetes

 For induction of type 1diabetes, rats were fasted overnight and received streptozotocin (60 mg/kg/d, i.p., dissolved in 0.1 mol/L citrate buffer, pH 4.5,Sigma, St. Louis, Missouri, USA). 3 days after the injection, the rats with fasting plasma glucose above 300 mg/dL (16.67 mmol/L) were classified as diabetic_._^24^

###  Exercise protocol

 The whole training period was 4 weeks. At first, the animals in the Exe group were adjusted to the animal treadmill. The examinations were conducted between 9 and 12 AM. The training program illustrated in the Table 1 that considered as mild training.^25^

**Table 1:** Training program

**Training program**	**Duration of training (min)**	**Speed (m/min)**	**Inclination of treadmill (degree)**
Fist week	day 1th: 10 min day 2th: 15 min day 3th: 20 min day 4th: 25 min day 5th: 30 min day 6th: 30 min day 7th:30 min	17 m/min	0
Second week	30 m/min	17 m/min	0
Third week	30 m/min	17 m/min	0
Fourth week	30 m/min	17 m/min	0

###  Elisa

 After treatment for one month, all rats were anesthetized with ketamine (80 mg/kg) and xylazine (5 mg/kg). Heart tissue was removed immediately and the left ventricle was used for measurement of protein levels of SIRT-1 and NOX4 by high-sensitivity ELISA kits (Zelbio, Germany). According to the manufacturer’s instructions, 100 mg samples were homogenized in 1 mL potassium phosphate buffer (pH 7.2 to 7.4) containing anti-protease cocktail for preventing of protein destruction. Then, the homogenate was centrifuged for 20 minutes at 4°C at 1000 ×*g*. Finally, the resulting supernatant was depleted and target proteins were extracted for ELISA analysis.

###  Blood glucose and HbA1c assessment 

 The HbA1c test, is an important blood test that gives a good indication of controlled diabetes condition. For this test, heart-collected blood sample was utilized to measure rat HbA1c levels in HbA1c assay kit (Crystal chem, cat number: 80300).

###  Immunostaining for PECAM-1/CD31

 To evaluate degree of vascularization in the heart, samples of left ventricle were used. So, 4 μm-thick slices were prepared from the samples. In the next step, these sections underwent processing steps such as deparaffinization, dehydration. To suppress endogenous peroxidase activity, incubation with proteinase K and treatment with hydrogen peroxide 0.3% were applied.

 The primary antibody CD31 (Santa Cruz, USA) was used as an indicator of angiogenesis to cover the samples. Later on the sections were incubated at 4°C for 12 hours. Then hatched with usual avidin–biotin complex (ABC; Santa Cruz) giving to the manufacturer’s orders. In the next step, light microscope (Olympus BX 40, Japan) was used for evaluating the strength scoring for CD31 staining (at magnification 40×). For this purpose, 3 to 5 1 mm^2^ areas were selected and staining strength each (at 200×magnification) and amount of positive cells were calculated. As a distinct container of the positive endothelial cell batch of immunoreactivities in interaction alongside with the elected region (1 mm^2^) was calculated. The granulated tissue was used as a positive control to measure the proportion of immunostaining, and the strength of staining was scored as 0 ( < 10%); 1 (10% to 25%); 2 (25% to 50%); 3 (50% to 75%) or 4 (75% to 100%).^26^

###  Statistical analysis

 Values are presented as mean ± SEM and were subjected to ANOVA followed by post hoc test (SPSS software version 16). Any *P *value < 0.05 was considered statistically significant.

## Results and Discussion

###  IGF-1 and exercise decrease HbA1c levels in the blood 

**Figure 1:**
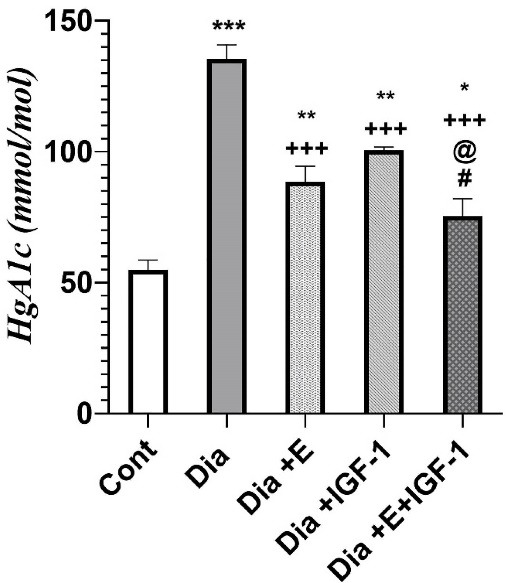
Effect of exercise and IGF-1 on HbA1c levels in the blood. Values are expressed as Mean ± SD. *P < 0.05,**P < 0.01,*** P < 0.001 vs control group. + + + *P* < 0.001 vs Dia group. @ *P* < 0.05 vs Exe group. # *P* < 0.05 vs IGF-1 group.

 According to Figure 1, HbA1C levels were found to be much higher (*P* < 0.001) in the diabetic subjects compared to the control group. Four weeks exercise and IGF-1 treatment in the Dia + E and Dia + IGF-1 groups significantly decreased HbA1c level (*P* < 0.001) in comparison to diabetes group. Meanwhile it was found to be noticeably (*P* < 0.01) higher compared to the control group. Moreover, simultaneous treatment with IGF-1 and exercise considerably decreased HbA1c compared to that in the Dia (*P* < 0.001), Dia + E (*P* < 0.05) and Dia + IGF-1 (*P* < 0.05) groups. According to Figure 1, combination therapy had a synergistic effect on HbA1c levels in the blood. Today, many studies has discovered that vascular complication in diabetes is mediated by hyperglycemia.^27^ As a result, the use of HBA1C reducing mediators in the treatment of diabetes-related heart failure has received a lot of interest. Many studies have demonstrated IGF-1’s capabilities in lowering blood glucose.^28^ Thus far, diabetic patients have shown to have lower IGF-1 levels in comparison to non-diabetic patients.^28^ Additionally, today, the improving role of physical activity in diabetes and its importance in regulating glucose hemostasis has been proven.^29^

###  IGF-1 and exercise improve NOX4 and SIRT-1 protein levels in the cardiac tissue 

**Figure 2:**
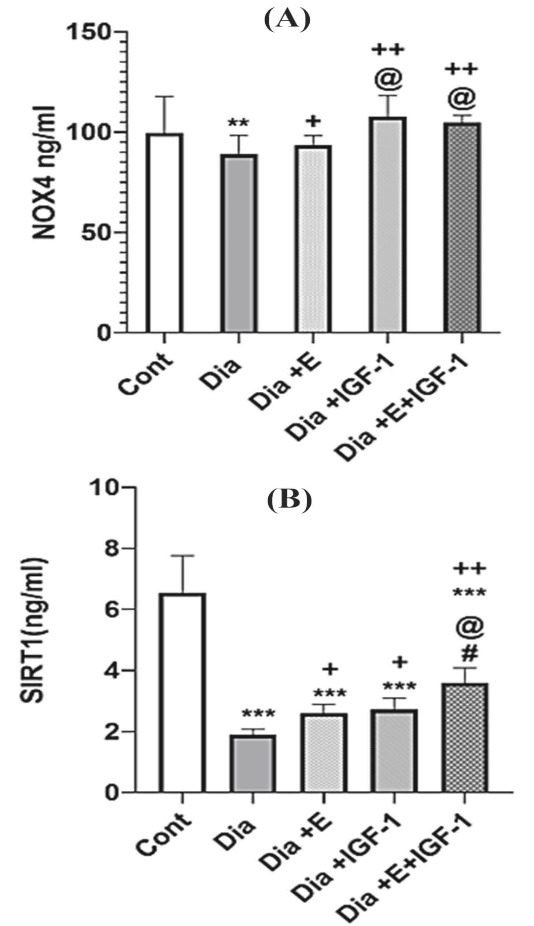
**
(A-B)** Effect of exercise and IGF-1 on NOX4 and SIRT-1 protein levels in the heart. Values are expressed as mean ± SD. **P < 0.01, *** P < 0.001 vs control group. + *P* < 0.05, + + *P* < 0.01, + + + *P* < 0.001 vs Dia group. @ *P* < 0.05 vs Exe group. # *P* < 0.05 vs IGF-1 group.

 The results achieved by ELISA indicated that diabetes in the early stage, remarkably reduced NOX4 (*P* < 0. 01) and SIRT-1 (*P* < 0. 001) protein levels in cardiac tissue comparing to that of the control group. As demonstrated in Figure 2 A-B, the four-week treatment of the diabetic rats with exercise remarkably (*P* < 0.05) grew NOX4 and SIRT-1 protein levels compared to that in the Dia group. Also, Figure 2 shows that IGF-1 therapy noticeably increased NOX4 (*P* < 0. 01) and SIRT-1 (*P* < 0. 05) protein levels in comparison to that in the Dia group. Interestingly, exercise and IGF-1 have been able to increase the NOX4 protein level similar to the control group but in regarding to SIRT-1, it was still significantly lower than that of the control group (*P* < 0.001). Moreover, the simultaneous use of exercise and IGF-1 caused an up rise in cardiac NOX4 level in contrast to Dia (*P* < 0.01), Exe (*P* < 0.05) groups. Nevertheless, according to the Figure 2B, in rats with the combination treatment of IGF-1 and exercise did not show any synergistic effect on NOX4 protein level. It seems that the increasing effect of combination therapy on the NOX4 protein levels mediated by IGF-1 and IGF-1 has a more potent effect on NOX4 protein level than exercise. In regarding to SIRT-1, simultaneously treatment with exercise and IGF-1 crucially raised SIRT-1 protein levels compared to Dia (*P* < 0.01), Dia + E (*P* < 0.05), Dia + IGF-1 (*P* < 0.05). It is cleared that combination therapy showed a synergistic effect on SIRT-1 protein levels in the heart tissue. Between a large numbers of molecular intermediaries associated to angiogenesis process, mediators such as NOX4 ^6^ and SIRT-1^30^ also show vital effect in adjust of new vessel development in the diabetes condition. Each of NADPH oxidase (NOX) isoforms are as major sources of ROS in the vessel wall. It is shown thatNOX2 and NOX4 are widely expressed in the heart.^31^ In this study, we focused on the function of NOX 4 molecule in the myocardium that is less well-understood. Recently, it is widely accepted that NOX4 is a significant source of ROS production in human endothelial cells.^32^ Also, molecular studies indicate that NOX4 stimulates angiogenesis signaling pathway through production of ROS, activation of tyrosine kinases receptor and the downstream extracellular signal-regulated kinase (ERK) pathway.^33^ In other hand, it is reported that in some pathological conditions, too much NOX-dependent ROS production, which is related with the over expression of distinct NOX isoforms, disrupt the redox control systems and induce oxidative injury of the cardiovascular cell.^34^ As, increased expression of NOX4 has been reported in hearts from diabetic model by Maeda et al in 2012.^35^ While, in this study, our results suggest that NOX4 protein expression declined in the heart of rats with diabetes and exercise and IGF-1 improved this reduction.

 In regards to the role of NOX4 in cardiomyopathy, Guzik et al showed that NOX-derived ROS has concerning the progression of vascular disease in diabetic patients.^36^ It seems that the role of NOX4 in pathology condition is arguable as specified by studies both reporting helpful^37^ and harmful effects^38^ of NOX4 in experimental heart failure. Hansen et al suggested that these discrepancies are due to the severity of the HF applied in the different studies.^39^ As, NOX4 may moderate advantageous impact via escalated angiogenesis in the development of a less serious heart failure in diabetic models.^39^ According to the results of the present study and previous research,^40^ it seems that the expression of NOX4 in the cardiomyocyte decreases in the beginning stage of diabetes but increases in the latter stage of diabetes. For example, Ebrahimian et al^40^ showed mice that were diabetic for two months had an increasing levels of NOX4, whereas our study showed rats that were diabetic for one month had a decreased levels of NOX4. The current study’s findings are consistent with those of a previous study demonstrating thatrats with diabetes for 4 weeks had increased levels of NOX4 mRNA in the heart but the protein expression of NOX4 did not show any increase.^41^ Lisa et al in 2011 indicated that subcutaneous injection of IGF1 in diabetic rats with myocardial infarction increased angiogenesis in cardiac tissue.^42^ The findings of our research also exhibited that IGF-1 injection rose angiogenesis in heart of diabetic rats. Humpert et al in 2008 displayed that insulin increases angiogenesis in endothelial cell through IGF-1 receptors.^43^ IGF-1 is also thought to promote angiogenesis by engaging with regionally generated factors like VEGF.^44^ In this study, histopathological assessment confirmed that IGF-1 treated group has a noticeable angiogenic reaction in the heart in compared to untreated rats. Qiao et al showed that IGF-1 increase angiogenesis by activating the AKT/ERK pathway^45^ and Bakr et al displayed that IGF-1 increased angiogenesis by activating the PI3K/HIF-1a/VEGF-A pathway.^46^ In completing studies to elucidate the mechanisms used by IGF to induce angiogenesis, we have shown for the first time that IGF-1 exhibits its pro-angiogenesis effects by increasing NOX4, SIRT-1. The current study also displayed those levels of NOX4, SIRT1 and in comparison, to inactive rats, capillary density elevated in the exercised groups. In line with our work, it is reported that treadmill exercise increases angiogenesis in the heart of diabetic subjects. Furthermore, it is showed that exercise increases the expression of SIRT1 in the aorta of rats.^47^ Exercise has also been shown to induce angiogenesis in the retina of rats by increasing NOX4 expression.^48^

 Furthermore, IGF-1 and exercise had a synergistic effect on HbA1c, cardiac SIRT-1 expression, and on neovascularization. But the combination treatment did not show any synergistic effect on NOX4 protein levels and it seems that the increased levels of NOX4 in the combination therapy group are due to IGF-1.

###  IGF-1 and exercise increase Angiogenesis in the heart tissue

**Figure 3:**
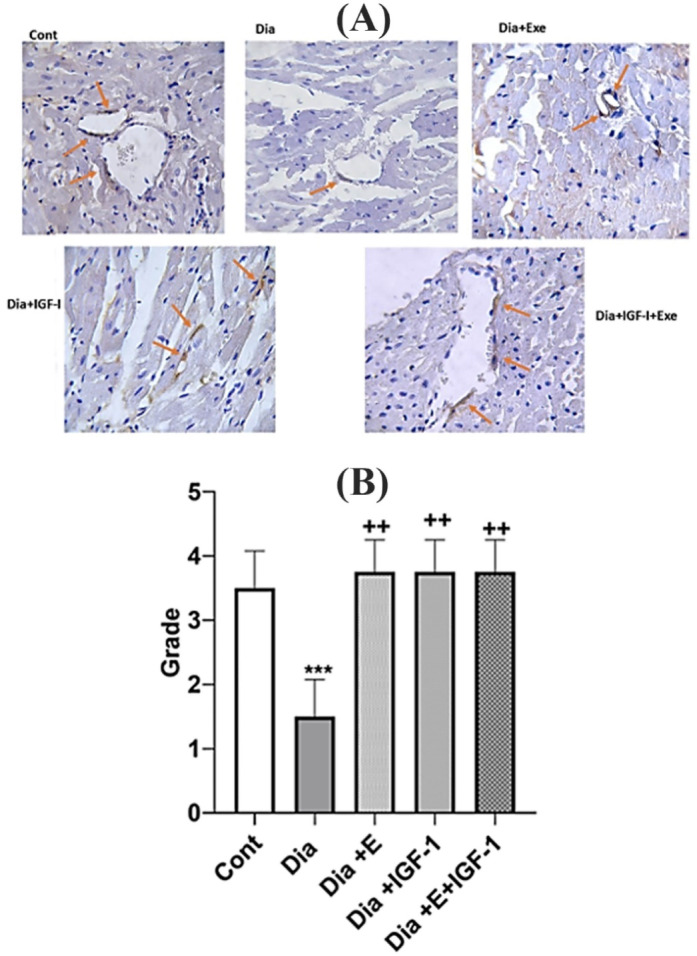
(**A**) Immunohistochemical detection of CD31 in heart. CD-31 immunostained endothelial cells were representative brown stained. A: Control; B: Dia; C: Dia + Exe, C:Dia + IGF-1, E: Dia + IGF-1 + Exe. Red arrows indicate the number and intensity of immunostaining for CD31. (**B**). Effect of exercise and IGF-1 on the angiogenesis in heart. Values are expressed as mean ± SD. ^***^ P < 0.001 vs control group. ^+ +^*P* < 0. 01 vs Dia group.

 Platelet endothelial cell adhesion molecule (PECAM-1) also known as cluster of differentiation 31 (CD31) is an extensively utilized marker of angiogenesis. In this study, the expression levels of CD31 were also evaluated by IHC (Figure 3A). The brown-stained tissues show CD-31 immunostained endothelial cells. Diabetes condition caused in a remarkably (*P* < 0.001) decreased angiogenesis compared to the control group. Each of exercise or IGF-1 had an increasing effect (*P* < 0.01) on angiogenesis and Dia + E and Dia + IGF-1 do not have any significant difference compared to control group. As, each of these interventions increased angiogenesis similar to that in the control group. Additionally, combination of IGF-1 and exercise in the Dia + E + IGF-1 group led into a noticeable (*P* < 0.01) rise in immunoreactivity for CD31 in comparison with Dia group. Statistical analysis of the immunohistochemically study showed that there was no notable rise in angiogenesis grade in the combination therapy group in comparison to each of Dia + E and Dia + IGF-1 groups. Actually, simultaneously treatment did not show any synergistic effects on angiogenesis (Figure 3B). Accumulating evidence showed that heart tissue in the diabetes condition displays functional changes that leading to myocardial dysfunction and cardiovascular diseases.^28^ Growing proof has shown that the progression of diabetes-related heart disorders was linked to a decrease in the development of new arteries.^29^ Therefore, a comprehensive description of the angiogenesis process and illumination of corresponding signaling pathways and involved molecules might be effective in the reducing heart complications in the diabetes condition. The outcomes of this study revealed that angiogenesis was reduced within the diabetic rat’s heart tissue. In addition, we showed that each of the exercise and IGF-1 interventions alone or in combination can increase the capillary density. In line with molecular results; histopathological comparison amongst the tissue of heart of the rats and the control group revealed a noticeable angiogenic reaction took place in the diabetic rats. We assume decreased angiogenesis in the diabetic rats mediated by decreased expression levels of NOX4. Probably with decrease levels of NOX4, activation of AKT and ERK proteins, which are the most important inducer angiogenesis, are also reduced. The angiogenic activity of endothelial cells is supervised extreme expression of SIRT-1 inside the vasculature throughout blood vessel development.^49^ Losing of SIRT1 function obstructs sprouting angiogenesis and bifurcation morphogenesis of endothelial cells with subsequent down-regulation of genes involved in blood vessel development and vascular renovation.^50^ Our results also showed that parallel with the decreased angiogenesis in heart tissue of diabetic rats, there was, a decrease in SIRT-1 expression levels in the heart of this group.^50^ In accordance to our study, Sulaiman et al^51^ in 2010 showed that SIRT1 decreased in the hearts of diabetic rats. Also, Huang et al showed that SIRT1 has a defensive impact on heart tissue.^50^ It has also been shown that SIRT1 increases angiogenesis responses in local ischemia of heart tissue in mice and shows a protective effect by suppressing NF-KB and increasing NO production in blood vessels.^52^ It has also been reported that sirt1 activates the MAPK/ERK and PI3K-Akt pathways, the two major signaling pathways involved in angiogenesis, and increases neovascularization.^13^

## Conclusion

 As a result, we showed that angiogenesis, NOX4 and SIRT1 protein levels decrease in the cardiac tissue in the early stage of diabetes. Also, IGF-1 and exercise could raise neovascularization in the diabetic rats maybe by increasing NOX4 and SIRT1expression.Additionally, the combination of IGF-1 and exercise have an improving synergistic impact on HgA1C and SIRT1 levels in the heart tissue of the diabetic rats in the early stage compared with each one alone. As a corollary to these findings, IGF-1 supplementation and exercise training may be considered as new therapeutic strategies for cardiomyopathy in diabetes condition. However, more studies are needed to elucidate the effective role of NOX4 in diabetic cardiomyopathy.

## Acknowledgments

 Financial support for this investigation by the Faculty of Medicine, Urmia University of Medical Sciences through grant is gratefully acknowledged.

## Author Contributions

**Conceptualization: **Leila Chodari, Shiva Roshan Milani.

**Data curation: **Saman Daneshfar, Bagher Pourheydar.

**Formal Analysis:** Leila Chodari, Bagher Pourheydar.

**Funding acquisition: **Leila Chodari.

**Investigation: **Shiva Roshan Milani.

**Methodology:** Leila Chodari,Bagher Pourheydar.

**Project administration: **Leila Chodari.

**Resources: **Saman Daneshfar.

**Software:** Leila Chodari.

**Supervision: **Leila Chodari.

**Validation:** Leila Chodari.

**Visualization: **Bagher Pourheydar.

**Writing – original draft:** Leila Chodari.

**Writing – review & editing:** Leila Chodari, Shiva Roshan Milani.

## Ethical Issues

 The animal care and experimental procedures were carried out according to the Guide for the Care and Use of Laboratory Animals published by the US National Institutes of Health (NIH publication no. 85-23, revised 2011) and the ethical principles under which the journal operates, and our work complied with this animal ethics checklist. The study was approved by the Animal Ethics Committee of the Urmia University of Medical Sciences, (ethical code: IR.UMSU.REC.1397.150). We have taken all steps to minimize the pain and suffering of the animals

## Conflict of Interest

 The authors declare that they have no conflict of interest.
